# Passinho RS, Bressan J, Hermsdorff HHM, Oliveira FLP, Pimenta AM. The 30-year cardiovascular risk trajectories and their independently associated factors in participants of a Brazilian cohort (CUME Study). Cad Saúde Pública 2023; 39(9):e00041323.

**DOI:** 10.1590/0102-311XER041323

**Published:** 2023-11-10

Where it reads:

**Figure 2:**
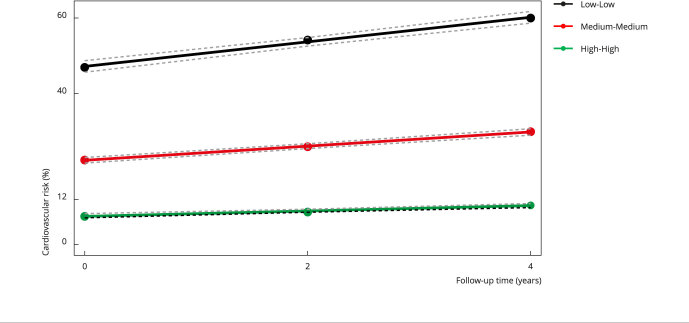
Trajectories of 30-year cardiovascular risk at four years of follow-up. Cohort of Universities of Minas Gerais (CUME Study), Brazil, 2016-2020.

Note: circles display observed values, whereas dotted gray lines represent 95% confidence intervals of adjusted trajectories. Low-Low = 68.3% of the total participants in this trajectory; Medium-Medium = 26.2% of the total participants in this trajectory; High-High = 5.5% of the total participants in this trajectory.

It should read:

**Figure 2:**
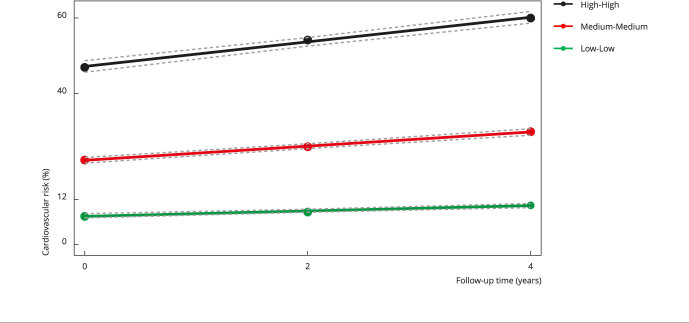
Trajectories of 30-year cardiovascular risk at four years of follow-up. Cohort of Universities of Minas Gerais (CUME Study), Brazil, 2016-2020.

Note: circles display observed values, whereas dotted gray lines represent 95% confidence intervals of adjusted trajectories. Low-Low = 68.3% of the total participants in this trajectory; Medium-Medium = 26.2% of the total participants in this trajectory; High-High = 5.5% of the total participants in this trajectory.

